# Molecular preservation in mammoth bone and variation based on burial environment

**DOI:** 10.1038/s41598-021-81849-6

**Published:** 2021-01-29

**Authors:** Caitlin Colleary, Hector M. Lamadrid, Shane S. O’Reilly, Andrei Dolocan, Sterling J. Nesbitt

**Affiliations:** 1 Department of Vertebrate Paleontology, Cleveland Museum of Natural History, Cleveland, OH 44106 USA; 2 Department of Geological Sciences, University of Missouri, Columbia, MO 65211 USA; 3 School of Earth Sciences, University College Dublin, Dublin 4, Ireland; 4 Texas Materials Institute, University of Texas at Austin, Austin, TX 78712 USA; 5 Department of Geosciences, Virginia Tech, Blacksburg, VA 24061 USA

**Keywords:** Palaeontology, Environmental chemistry

## Abstract

Biomolecules preserved in fossils are expanding our understanding of the biology and evolution of ancient animals. Molecular taphonomy seeks to understand how these biomolecules are preserved and how they can be interpreted. So far, few studies on molecular preservation have considered burial context to understand its impact on preservation or the potentially complementary information from multiple biomolecular classes. Here, we use mass spectrometry and other analytical techniques to detect the remains of proteins and lipids within intact fossil mammoth bones of different ages and varied depositional setting. By combining these approaches, we demonstrate that endogenous amino acids, amides and lipids can preserve well in fossil bone. Additionally, these techniques enable us to examine variation in preservation based on location within the bone, finding dense cortical bone better preserves biomolecules, both by slowing the rate of degradation and limiting the extent of exogenous contamination. Our dataset demonstrates that biomolecule loss begins early, is impacted by burial environment and temperature, and that both exogenous and endogenous molecular signals can be both present and informative in a single fossil.

## Introduction

Molecular taphonomy, the study of how biomolecules persist and degrade in the rock record, has developed notably in the last few decades with the increased use of new technology (e.g., high-resolution mass spectrometry)^1–3^. Studying ancient DNA is now commonplace in archaeology and fossil material that is less than 1 million years old, but studies that seek to examine the molecular information of ancient animals on longer timescales have turned to biomolecules with greater preservation potential^4^. Therefore, studies on vertebrate fossils often focus on detecting structural biomolecules (excluding carbohydrates) that can be found in bone (e.g., proteins, lipids) and their degradation products (e.g., peptides, amino acids, advanced glycosylation end-products, free fatty acids, steranes, advanced lipoxidation products)^5–11^.

Proteins have been examined in fossil bone and contribute to data on i*n vivo* changes and evolutionary adaptations. A mammoth (*Mammuthus primigenius)* bone preserved in permafrost (~ 43,000 years old) yielded 126 proteins and 962 unique peptides, providing information on amino acid substitutions and protein damage that occurred while the animal was alive^12^. When the bone preserved in permafrost was compared to mammoth bones that were preserved in more temperate environments, considerably fewer proteins and peptides were recovered, suggesting that burial environment greatly influences molecular preservation, even on very short timescales^12^.

Despite studies that have observed preservation differences based on burial environment^9,13–15^, no study has yet compared biomolecule preservation across burial environments in closely related taxa. Therefore, we analyzed a dataset that includes a mammoth rib from permafrost, a rib from a channel deposit, a rib from a hot spring-fed sinkhole, and a rib from a natural asphalt sink, to examine changes in molecular preservation based on burial environment. We analyzed intact bone, without using extraction techniques, using surface mass spectrometry and Raman spectroscopy to understand the overall molecular taphonomy and preservation. We experimentally degraded elephant bone at low and high temperatures to compare the changes that occurred to the molecular signatures found in fossil bone. Additionally, we included sediment samples associated with two of the mammoth fossils as controls. Furthermore, we extracted and examined lipids (biolipids and their degradation products) because of their high preservation potential^16^ and established use as biological and environmental marker compounds in the rock record^4^.

## Results

### Amino acids

**Figure 1:**
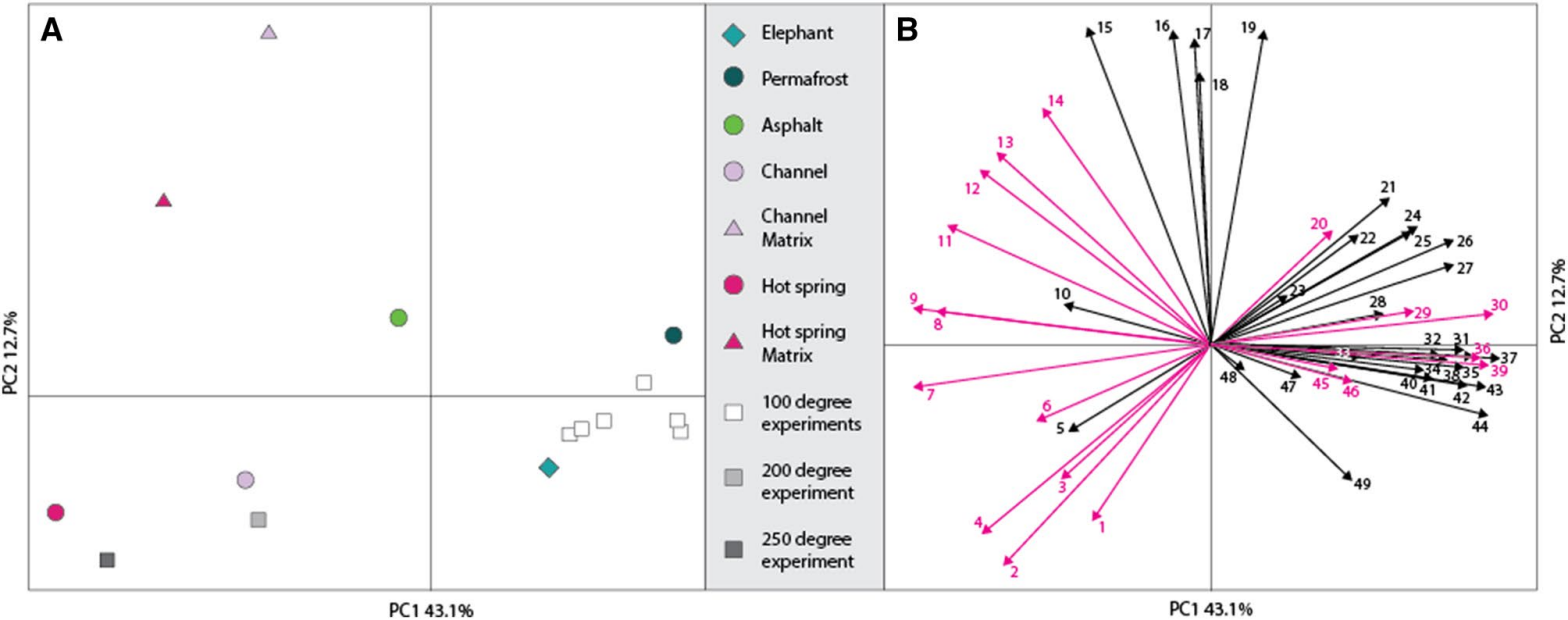
(**A**) Principal component analysis (PCA) of 49 peaks associated with amino acid fragments detected using TOF–SIMS. The modern elephant bone (open rhombus) plots near the permafrost mammoth fossil (open circle) and the low temperature maturation experiments (open square). The channel deposit and hot spring fossils (open circle) plot with the 200 °C and 250 °C high temperature maturation experiment (open square). The matrix (open triangle) and the asphalt mammoth fossil (open circle) plot separately. (**B**) Loadings that show the influence of the 49 peaks on the placement of the samples in the PCA space. The black arrows represent the peaks assigned from^24^ and the pink arrows are the new peaks assigned in this study. The amino acid fragment assignments are in Table S1.

Unaltered elephant bone, experimentally degraded elephant bone, and the mammoth fossils were analyzed using time-of-flight secondary ion mass spectrometry (TOF–SIMS). Based on the TOF–SIMS mass spectra, we established an amino acid signature (or fingerprint) made up of 49 amino acid fragments detected in all of the samples. We applied principal component analysis (PCA), a multivariate statistical method, to compare the samples. Figure 1 shows that > 55% of the variance between the samples is accounted for in principal components 1 (PC1) and 2 (PC2) (Fig. 1A). The corresponding loadings (Fig. 1B) show the influence of each amino acid fragment selected to represent the amino acid signature on the placement of the specimens in the PCA space. The fresh unaltered elephant bone has a similar amino acid signature to the bones heated to 100 °C and the mammoth bone preserved in permafrost. Meanwhile, the bones heated to 200 °C and 250 °C have signatures more similar to the mammoth bones from the channel and hot spring deposits. The sediment matrix samples taken from the channel and hot spring deposits act as controls and are distinct from the mammoth bones from these sites on the y-axis (PC2).

The mammoth rib from the asphalt is distinct from all fossil and sediment matrix samples, despite good organic preservation being reported from this burial environment^17^. The use of surface mass spectrometry on this fossil sample was restricted by the tar matrix contaminating the fossil, observed also in the heavy fluorescence appearing in its Raman spectrum. Solvent co-extraction of high concentrations of hydrocarbons in the tar matrix further restricted the application of lipid analysis.

### Amides and apatite

**Figure 2:**
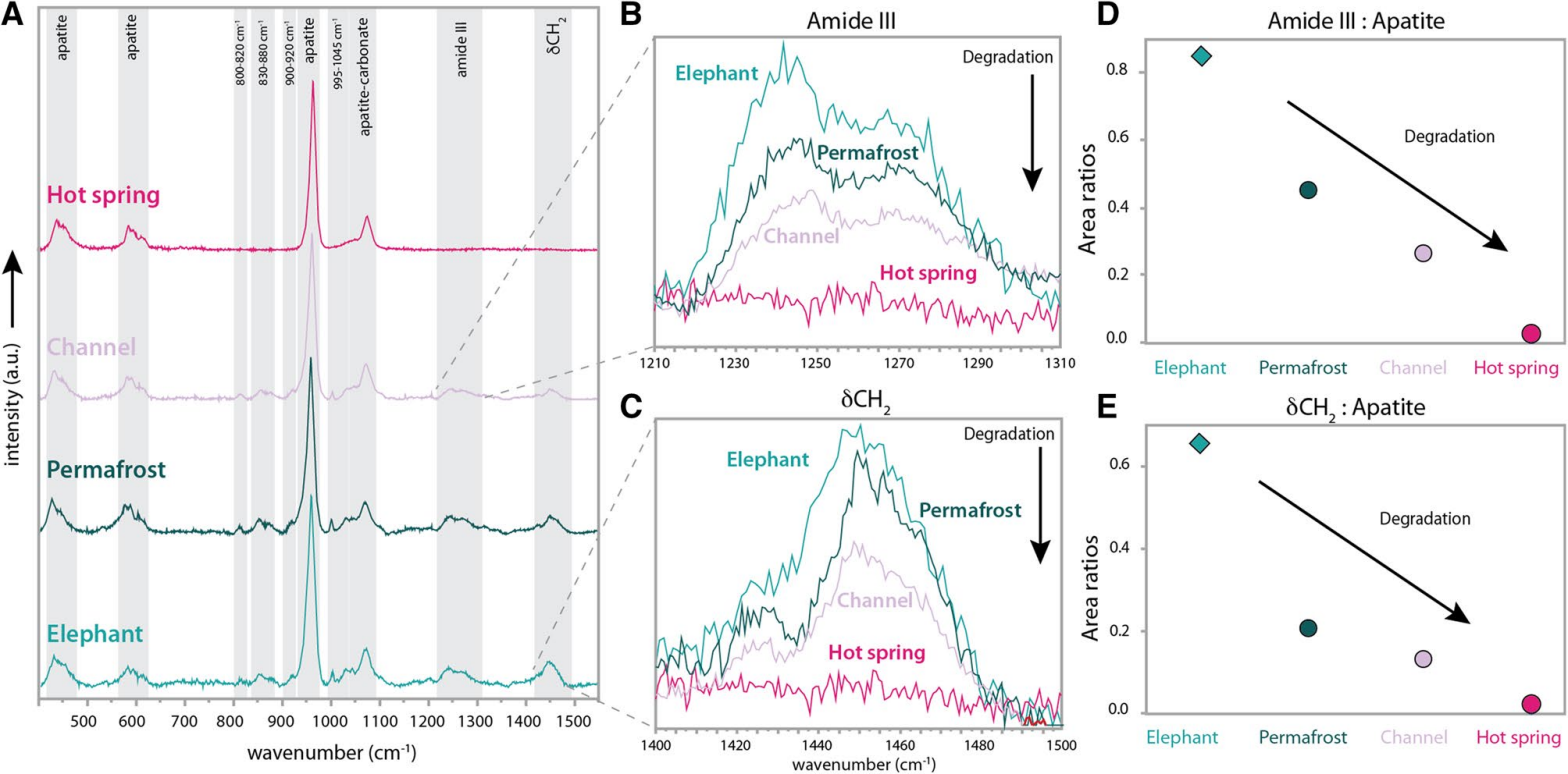
Representative Raman spectra of the mammoth fossilized bones, modern elephant bones and the degree of preservation of organic material. (**A**) Raman spectra of the mammoth fossils: hot spring (dark pink), the channel (light pink), permafrost (dark green) and extant elephant (light green). Highlighted are different vibrational Raman bands of the crystalline apatite and amides. (**B**) Close-up of amide III band showing degradation from the elephant bone to permafrost to channel to hot spring. (**C**) Close up of δCH_2_ band that shows the same pattern of degradation. (**D**) The ratio of the amide III band to apatite and (**E**) the ratio of δCH_2_ band show the same pattern of degradation.

**Figure 3:**
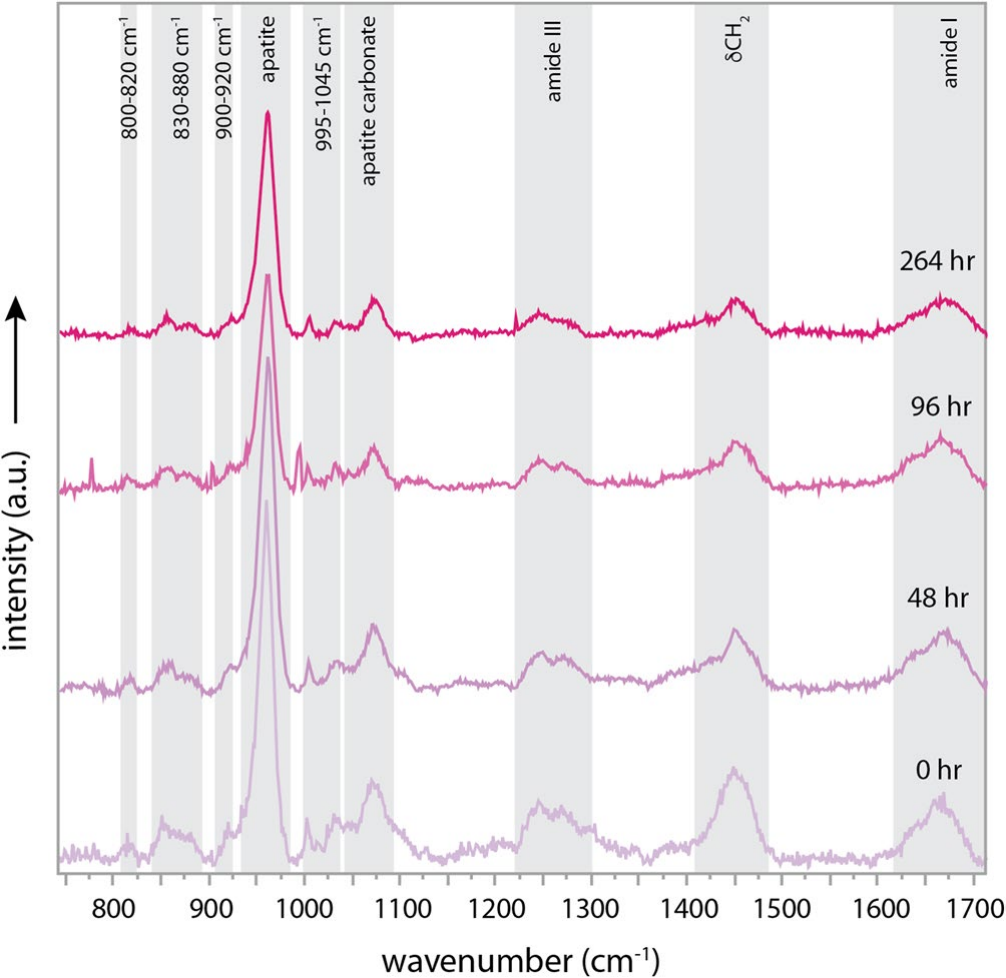
Raman spectra of the maturation experiments of the modern elephant rib bone at 100 °C 264 h (11 days). Degree of degradation increases with time and it is exemplified by the decrease in the relative sizes of the main amide bands (orange) amide III ~ 1225–1300 cm^−1^, the C–C stretching band ~ 1410–1480 cm^−1^ and amide I ~ 1650–1700 cm^−1^ in comparison to the apatite bands (highlighted).

Raman spectra obtained from the mammoth fossils show similar Raman bands in comparison to the unaltered elephant bone (Figs. 2A and 3). The different vibrational modes of the mineral structure of bone (apatite) are the most prominent peaks in all the samples (peaks at 960, 1070, ~ 590 and ~ 450 cm^−1^), followed by different Raman bands that have been previously assigned in the literature to organic molecules^18–20^ (amide III between 1220 and 1300 cm^−1^; δCH_2_ between 1410 and 1480 cm^−1^; amide I between 1620 and 1700 cm^−1^), along with other smaller organic peaks corresponding to AGEs^9^ (see Fig. 2A and Table S2). It is important to note that the Raman bands that correspond to different vibrational modes of different organic molecules (proteins, lipids, etc.) will be located close to each other in the same frequency region. The vibrational frequencies of the C–C, C–H or C–N symmetric stretching, for example, will be located very close for different molecules. As such, the peaks assigned to organic molecules represent an amalgamation of the vibrational frequencies of different organic molecules that will form a large set of peaks.

The apatite Raman bands are strong in all of the fossils, whereas the organic peaks show different intensities under the same collection times and can be used as proxies for different degrees of organic molecule degradation (Fig. 2B,C). We conducted semi-quantitative analyses of the Raman peak areas by normalizing the organic peak areas to the peak area of the main apatite peak (ν_1_ PO_4_^3−^ at 960 cm^−1^) of each fossil (Fig. 2D,E). The trends show that the organic molecules corresponding to the permafrost and channel deposit fossils have more organic peaks present than the hot spring fossil, where none of the organic peaks were observed. Similar results were observed in the experimentally matured elephant bone. The same bone fragment was observed at different times after setting the bone at 100 °C. The Raman spectra show that the areas under the peaks of the organic molecules (amide I, δCH_2_, and amide III) become slightly smaller with time (Fig. 3). The mammoth fossil preserved in asphalt was not suitable for Raman studies because the hydrocarbon impregnation of the sample led to severe fluorescence, obscuring the Raman scattering of the sample.

### Lipids

**Figure 4:**
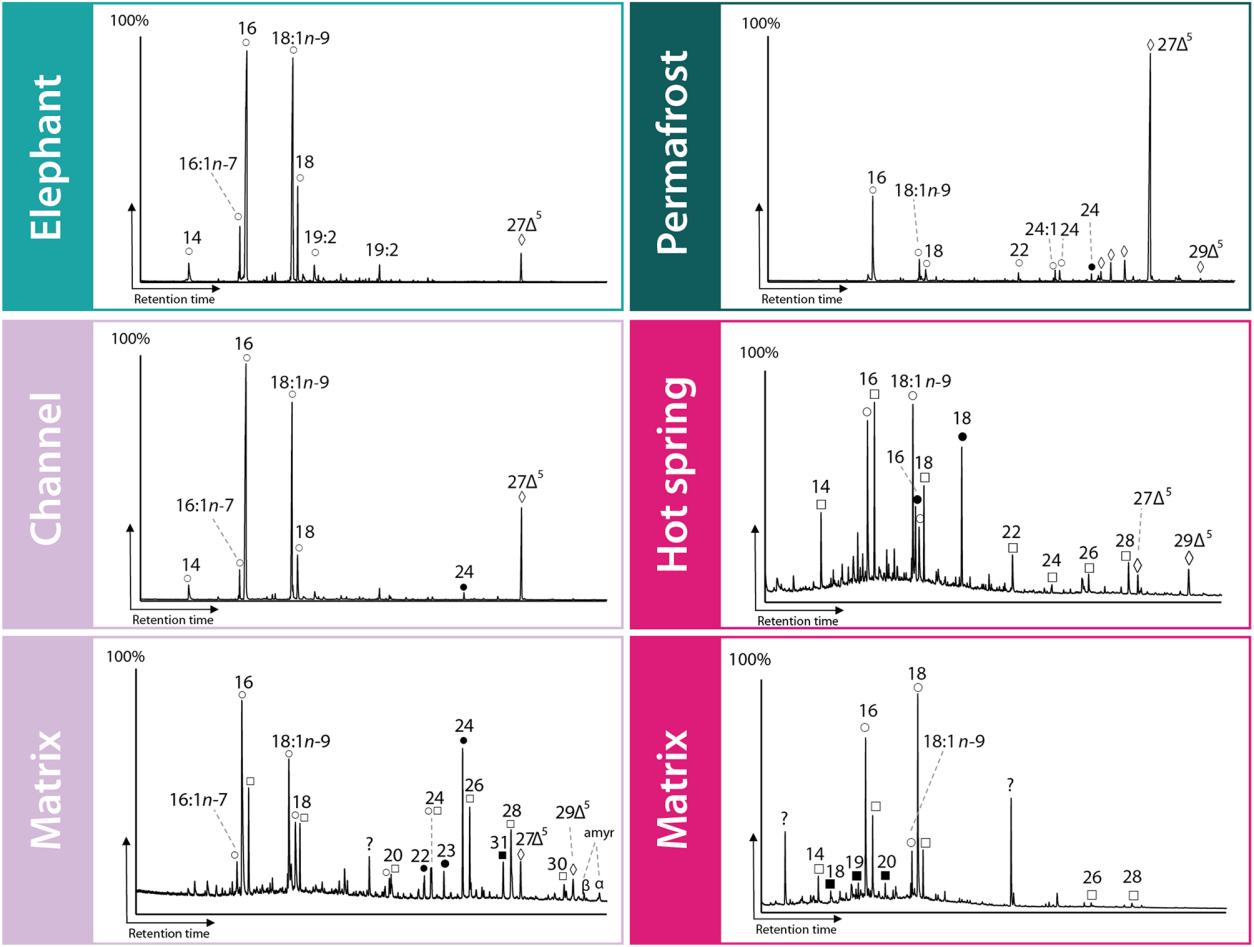
Partial total ion chromatograms showing the lipid distributions from extant African elephant bone, mammoth bones from permafrost, channel deposit and hot spring-fed sinkhole and associated matrix from the channel and hot spring deposits. ‘open circle’ are fatty acids, ‘filled black circle’ are 2-hydroxy fatty acids, ‘open square’ are n-alkan-1-ols, ‘filled black square’ are n-alkanes and ‘open rhombus’ are sterols. ‘amyr’ corresponds to alpha and beta amyrin.

The major detected lipids in this study were saturated and monounsaturated fatty acids, sterols, *n*-alkan-1-ols, *n*-alkanes, pentacyclic triterpenoids and 2-hydroxy acids. Lipids in vertebrate bones are derived primarily from marrow adipose tissue and dominated by hexadecanoic acid (16:0), oleic acid (18:1n-9), octadecanoic acid (18:0), tetradecanoic acid (14:0) and cholesterol (cholest-5-en-3β-ol; 27Δ^5^)^21,22^. This is confirmed here from the analyzed unaltered elephant bone (Fig. 4, Supplement Figure S2). The channel deposit fossil and extracted outer surface material were dominated by 16:0, 18:1n-9 and 18:0 fatty acids and cholesterol (cholest-5-en-3β-ol; 27Δ^5^) (fossil interior shown in Fig. 4). The fossil bone also contained appreciable 16:1n-7 and 14:0. The sediment matrix contained a more diverse lipid profile with significant amounts of higher plant-derived long chain (greater than 20 carbon chain length) 2-hydroxy acids, *n*-alkan-1-ols and *n*-alkanes and higher plant pentacyclic triterpenoids α-amyrin (urs-12-en-3β-ol) and β-amyrin (olean-12-en-3β-ol)^22,23^. Higher plants lipids are usually the major extractable lipids in soils and riverine settings, reflecting the dominant input of plant material to organic matter in these environments^22,23^.

The hot spring fossil contained most lipids in the cancellous bone and only trace levels of lipids in the compact bone (Figure S3) and comprised a mixture of fatty acids, *n-*alkan-1-ols and 2-hydroxy acids with 16 and 18 carbon chains lengths. Lower relative amounts of long chain *n-*alkan-1-ols, 27Δ^5^ and β-sitosterol (24-ethylcholest-5-en-3β-ol; 29Δ^5^) were also observed. Sterols and 2-hydroxy acids were not detected in the sediment matrix. The fossil surface and sediment also contained a number of unidentified compounds as major compounds. The permafrost fossil compact and cancellous bones both contained 27Δ^5^ as the major lipid, as well as a number of 27Δ^5^ derivatives (Fig. 4). The cancellous bone contained plant-derived long chain *n*-alkanols, 2-hydroxy acids, *n*-alkanes, 29Δ^5^, α-amyrin and β-amyrin in high amounts relative to other samples.

## Discussion

To examine the molecular taphonomy of mammoth bone, we used surface mass spectrometry and spectroscopy (TOF–SIMS and Raman) to collect amino acid and amide data that indicate protein preservation and bone degradation, and we compared that to lipid data that reflects both preservation and contamination. Protein preservation is well documented in fossil bone of this age^2,12,13,24^, therefore we chose a sampling of fossils from different burial environments (i.e., permafrost, a channel deposit, a hot-spring fed sinkhole and an asphalt sink) to compare variation in preservation. If endogenous biomolecules are to be found in fossils, preservation must begin very quickly, during early diagenesis^13^. There are many factors that impact degradation, including temperature, pressure, fluid interactions and oxygen levels^13–15,25^. We conducted a series of bone degradation experiments on modern elephant bone focusing on a single diagenetic variable—temperature—to evaluate its impact on the amino acid signature and examine how the altered bone compares to what has occurred in the fossils. Experiments that thermally break down chemical bonds to simulate degradation are commonly used to study kerogens and coal but are now being used more often to study the breakdown of macromolecules in biological samples^26^.

To determine the relationship between samples we constructed an amino acid signature consisting of 49 amino acid fragments (Fig. 1), assigned based on the atomic mass of their corresponding peaks in the TOF–SIMS mass spectra (Fig. 1B in pink). This signature also includes peaks assigned in a previous study^24^ (Fig. 1B in black, Supplement Table S1). We used this combination of peaks to best represent our specific samples within the context of previous work evaluating amino acid preservation in bone using TOF–SIMS. Unaltered and thermally matured elephant bone were included in the analysis, with the fossil samples and two associated matrix samples. We applied multivariate statistics (i.e., PCA) on the amino acid signature dataset extracted from the TOF–SIMS mass spectra. The majority of the statistical variation appears along the first principal component (PC1 43.1%) with the unaltered elephant bone, the elephant bone heated to 100 °C and the permafrost mammoth bone grouping together and with the mammoth bones from the channel deposit and sinkhole plotting with the elephant bones heated to 200 °C and 250 °C. The y-axis (PC2 12.7%) demonstrates the majority of the variation between the fossils and the sediment matrix controls. Therefore, the selected amino acid signature distinguishes three groups: (1) the modern elephant bone, the low temperature experiments and the permafrost mammoth bone, (2) the channel deposit and sinkhole mammoth bones and the higher temperature experiments and (3) the sediment matrix controls. The mammoth bone from the asphalt sink is not being considered in our interpretation because we found that it was heavily contaminated with organics from the burial environment that cannot be separated from amino acids signatures using surface mass spectrometry. Despite these three distinct groups, the placement of the modern elephant bone is unexpected, demonstrating that this method cannot distinguish between protein degradation products that are unique to bone and those that are from the environment. The amino acid signature does however record changes from thermal degradation and a strong variation between the mammoth fossils from the channel and hot spring deposits and their associated matrix. Therefore, amino acid data using surface mass spectrometry should be used in conjunction with additional chemical techniques to further discern information that is complicated by diagenesis.

The amino acid findings are complemented by the data from the Raman analyses, which demonstrate degradation based on the loss of the amide III and δCH_2_ bands (Fig. 2). The degradation of organic molecules in the bone structure can be increased or slowed down as a function of bone density by the shielding provided by the apatite crystal (see Fig. in^27^ for different bone densities). Semi-quantitative analyses of the Raman bands ratios of the organic peak areas (δCH_2_, and amide III) in the experimentally matured elephant bone (Fig. 3) normalized against the peak area of the main apatite band (960 cm^−1^) for each Raman spectra shows that the level of degradation of the organic peak increases in the less dense bone areas of the elephant bone fragment (inner cortical bone) and is greater in the higher density area of the external cortical bone (Figure S1).

We examined specific areas of intact fossil bone to better understand how molecules may preferentially preserve in certain locations and how this information is impacted by environmental contamination. The thermal degradation of the bone also affects the mineral structure of the apatite (Figure S2). The peak position of the apatite mineral shifts towards higher wavenumbers in all the different parts of the bone fragment, including the cancellous bone, and inner and external cortical bone (reported in^28–30^). Moreover, the width of the apatite peak (FWHM) also increases significantly more for the cancellous bone than for the inner cortical bone and the external cortical bone. This suggests that the degree of degradation of the porous bone is higher, and the broadening of the peak (FWHM) is explained by the appearance of an increased number of smaller apatite crystals^30^.

We also assessed the preservation state of endogenous lipids and extent of exogenous contamination from the lipid data according to the following criteria: (1) fossils matching the occurrence and relative abundances of lipids from elephant bones are likely preserved endogenous signals, and (2) fossils with considerable amounts of lipid sourced from soil plant matter and bacteria^22,23^ and/or similar lipids as sediment matrices are likely significantly contaminated with exogenous organic matter. If significant contamination from soil, either particulate or aqueous dissolution and transport, has occurred, then vascular-plant lipids such as α/β-amyrin, 29Δ^5^ and plant wax long chain fatty acids, *n*-alkanols, hydroxyl acids (with even carbon number predominance) or *n*-alkanes (odd carbon chain predominance) would be found^25,32^. Bacterial contamination of the fossil bones would be suspected if bacterial fatty acids such as methyl-branched C15 and C17 fatty acids and/or bacterial hopanoids^22^ were abundant.

Based on these criteria we conclude that the sediment matrix from the channel deposit was dominated by soil organic matter, mainly coming from plant litter. This conclusion is consistent with the freshwater stream depositional and environmental setting. However, negligible amounts of plant lipids were detected on the surface of the fossil or from the fossil itself, indicating very little contamination and suggests that that the observed lipids in the fossil are endogenous. The lipid profile from the channel deposit fossil matches the expected profile for vertebrate bone lipids. In addition, the occurrence of lipids with labile unsaturated bonds (18:1n-9 and 27Δ^5^) indicates exceptional preservation and little early diagenetic alteration. Thus, despite the age (37 ka), the fossil from the channel deposit appears to have exceptional preservation of endogenous biolipids.

In contrast to the channel deposit, the sediment matrix that hosted the hot spring fossil did not contain a plant litter signal. The occurrence of hexadecan-1-ol, octadecan-1-ol as major lipids, together with *n*-alkanes between C18 and C20 indicates a microbial, likely microalgal, source of organic matter^31^. While 16:0, 18:1n-9, 18:0, 14:0 and 27Δ^5^ were present, the fossil also contains appreciable microalgal lipid signals. This indicates contamination of the fossil with exogenous lipids from microalgae thriving in the hot-spring. Notably, the compact bone contains low abundances of lipids relative to the cancellous bone and sediment matrix. A progressive, increasing concentration gradient of organic molecules from matrix to the center of a fossil (or rock) provides the best evidence for endogenous and syngenetic signals^32^. Given the concentration gradient found for the hot spring site, together with the co-occurrence of lipids, it is possible that inward diffusive migration of lipids sourced from aquatic algae in the hot spring have contaminated the fossil over time.

The permafrost fossil compact and cancellous bones both contained 27Δ^5^ as the major lipids, as well as 16:0, 18:1n-9, 18:0 as the major fatty acids. Compared to the modern elephant bone, 27Δ^5^ is much more abundant than the fatty acids. The progressive loss of fatty acids relative to 27Δ^5^ agrees well with expected degradation rates for these lipid classes^33^. However, both the cancellous and compact bone also contained vascular plant signals, particularly in the cancellous bone. While a sediment control sample was not available for this fossil sample, it appears that significant contamination of the cancellous bone has occurred and to a much lower degree for the more external compact bone. For both the permafrost site and the hot spring site, the higher relative abundance of lipids in the cancellous bone compared to the cortical bone is likely due to the higher porosity of cancellous bone and increased susceptibility to diffusive contamination. Therefore, we recommend that cortical and cancellous bone should be analyzed separately in lipid studies of fossil bones, and that, generally, cortical bone is the more likely to better preserve endogenous signals and be less contaminated. In general, there was no clear influence from bacterial lipids such as methyl-branched short chain fatty acids or hopanoids, indicating that extensive bacterial colonization and contamination did not occur.

The preservation of endogenous lipids in archaeological and fossil remains of various vertebrates^10,22^ has been reported, but information on their depositional context have rarely been assessed and compared. Based on our lipid data, the fossil preserved in the hot spring shows the poorest preservation and the highest degree of contamination. The channel deposit fossil appears to show exceptional preservation of endogenous lipids and little evidence of exogenous organic contamination. Given the age of the permafrost fossil, the detection of well-preserved biolipids (particularly 27Δ^5^) with very little exogenous contamination (compact bone) is remarkable. We attribute the poor preservation of the hot spring fossil to the contrasting depositional environment, whereby the higher temperatures that the fossils in the hot-spring fed sinkhole were subjected to would result in a higher rate of decay of more labile lipids. The microalgal signal that dominates the sediment matrix and that was also observed in the fossils could result from colonization by aquatic microalgae growing in the hot spring. Thus, enhanced degradation of endogenous organic matter in this setting is likely. Because the grade of preservation does not precisely corelate directly from the age of the fossils, the depositional and environmental conditions are the primary factor influencing preservation. In particular, as for DNA, temperature appears to be a major parameter influencing preservation of lipids in vertebrate bones.

Considering the age of the fossil material analyzed, the presence of biomolecules falls well within the timeframe for both protein and lipid preservation as determined previously^2,12,13,24^. The protein results, including the presence of amides and amino acids, correspond with apatite degradation and demonstrate that the permafrost bone is the best preserved, the hot spring bone has undergone the most degradation (both in the structure of the bone and at the molecular scale), and the bone preserved in the channel deposit falls between the two. Interestingly, the bone from the channel deposit has better lipid preservation than the permafrost fossil bone, indicating that molecular scale degradation varies based on both burial environment and the macromolecule being analyzed. Going forward, it is important to note from our study that preservation and contamination vary based on what part of the bone is sampled. Degradation occurs on the exterior portion of cortical bone at a higher rate than the interior and soil contaminants are detected less in the interior of the cortical bone. This should be carefully considered in future studies of molecular preservation and possibly in other studies extracting elements from boney tissues (e.g., isotopic). Furthermore, inclusion of lipid data also establishes that both exogenous and endogenous molecular signals can be present and informative in a single fossil sample and contributes another dataset to compare to protein preservation in extinct forms.

## Materials and methods

### Elephant and mammoth bones

Mammoth rib fragments from the Mammoth Site (07HS152), Shultz mammoth site (39MD900), Rancho La Brea (HC142067) and Canyon Creek, Yukon (YG546.52) were analyzed. A modern African elephant (*Loxodonta africana*) rib from a deceased zoo animal from the Mammoth Site collection that was never buried was analyzed at varying temperatures to test thermal degradation and make comparisons to the fossil specimens. The Mammoth Site of Hot Springs, South Dakota is a sinkhole deposit (laminated fine-grained sediment from clay to coarse sand^34^) that was heated by hot springs year round (~ 35 °C) and is currently dated at ~ 26 Ka (although based on new research may be as old as ~ 190 Ka^35^. The rib (07HS152) used in this study was buried at an estimated 7 m depth. The Schultz mammoth (39MD900) was excavated in a fine-grained stream channel deposit that was radiocarbon dated to ~ 37–39 Ka. Analyses of the site determined that the mammoth was buried in a low energy deposit and the rib used in this analysis was found in silt between two high-energy, gravel-filled gullies^36^. The Rancho La Brea mammoth rib fragment was excavated from Pit 9, which has a mean calibrated radiocarbon date of ~ 24.5 Ka^37^. The Yukon mammoth rib fragment was surface collected in Canyon Creek in 2014 and was originally preserved in permafrost. The age of this sample is not well-constrained because it was not collected in situ, however it can be estimated to be < 150 Ka (pers comm. Grant Zazula).

### Maturation experiments

A diamond saw (Dremel) was used to cut Modern African elephant rib bone into two sizes (2 mm and 1 cm) for the short (i.e., 24 h) and long (i.e., up to 91 days) term experiments. For the 24 h experiments, three 2 mm^2^ fragments were inserted into 3 × 15 mm platinum capsules and loaded by hand into cold-sealed pressure vessels at 100 °C, 200 °C, and 250 °C at atmospheric pressure (following a similar loading procedure to^38^). The five long term experiments were cut into 1 cm fragments and placed in an oven at 100 °C under vacuum at − 12.3 psi. The short-term experiments were terminated at 24 h, while the long-term experiments were terminated at 7, 14, 30, 67, and 91 days*.* An extra maturation experiment was conducted to analyze the effect of temperature (at 100 °C) in the same bone fragment (1 cm) using in situ Raman spectroscopy. Different parts of the structure of bone fragment (cancellous, internal cortical bone and the external cortical bone) were analyzed with the Raman microscope at 0, 1, 2, 3, 4 and 11 days. The bone piece was placed under vacuum at 100 °C and taken down for analyses and loaded again at 100 °C immediately after the Raman analyses.

### Time-of-flight secondary ion mass spectrometry (TOF–SIMS)

Time-of-flight secondary ion mass spectrometry (TOF–SIMS) was performed using an ION-TOF TOF.SIMS 5 at The University of Texas at Austin, Texas Materials Institute. A pulsed (20 ns, 10 kHz) analysis ion beam of Bi_3_^+^ clusters at 30-kV ion energy was raster-scanned over 500 × 500 μm^2^ areas. Bi_3_^+^ polyatomic sputtering was used to enhance the signal and reduce the fragmentation of large organic molecules. A constant flux, 21 eV electron beam was used during data acquisition to reduce sample charging. The detected secondary ions had positive polarity and an average mass resolution of 1–3000 (m/δm). The base pressure during acquisition was < 1 × 10^–8^ mbar. Mass calibration was performed by identifying the peak positions of CH_2_^+^, CH_3_^+^, O^+^, F^+^, Na^+^, K^+^, Ca^+^, and Cs^+^ secondary ions. Regions of interest were chosen to reduce the effects of topography. Of the amino acids analyzed, aspartic acid (Asp) was not included because it could not be separated from the Cs signal, which was used to shallow sputter each sample (133 amu) for adventitious contaminants removal.

Amino acids were detected and mapped in the bone in situ using TOF–SIMS in modern elephant and fossil mammoth rib bones. Amino acid assignments (Tables S1 and S2) were made based on molecular weight, the assignments of fragmented amino acids established in previous fossil studies^24^ and additional amino acid fragments shared between samples that were identified in this study. In total, we established an amino acid signature with 49 amino acid fragments for principal component analysis (PCA), to evaluate the variance between the samples, including the experimentally matured elephant bone and two sediment matrix samples from two of the mammoth sites (i.e., the channel deposit and sink hole) were analyzed as controls. The yields of the 49 peaks of interest were measured and normalized to their sum and standard deviation before running the PCA.

### Raman spectroscopy

Raman analyses were conducted using a JY Horiba LabRam HR (800 mm) spectrometer with a 600 grooves/mm grating in the Department of Geosciences at Virginia Tech. The confocal aperture was 400 µm, with a slit width of 150 µm. Excitation was provided by two lasers to circumvent fluorescence of the sample, a 632.9 nm HeNe (power of 20 mW at the source and ~ 2mW at the sample) and a 785 nm (a solid state-diode laser operating at ~ 150 mW). The Raman uses an air-cooled (− 70 °C) CCD detector with a 1024 × 256 pixels front illuminated chip and a 600 grooves/mm gratings. The laser was focused through a 10× objective with a working distance of ~ 15 mm from the sample surface. The best spectral data was achieved using no filter for 3 accumulations, with a collection time of 300 s. Areas of interest were also selected for fossil samples (400–1500 with the 632 nm laser and 2500–3300 with the 785 nm laser), which reduced background fluorescence. Labspec 6 software was used for spectral data reduction (baseline correction, peak fitting and peak intensity and areas) using a Gaussian/Lorentzian function to determine spectral features like peak position, area under the peak and full width at half maximum (FWHM). Raman band where assigned to different materials based on different published materials and are displayed in Table S2. Peak area ratios where conducted always normalizing the organic peaks (δCH_2_, amide III) to the apatite ν_1_ (PO_4_^3−^) peak.

### Lipid extraction and gas chromatography mass spectrometry (GC/MS)

Outer surfaces of fossil and modern bones were cleaned using a solvent-washed scalpel and where possible, this material was retained and treated as a distinct sample. Where possible (bone from the permafrost and hot spring), compact bone and cancellous bone were separated using the scalpel and extracted separately. Fossil and sediment matrix samples were powdered using a SPEX 8500 shatterbox and stainless steel puck mill. Between 1 and 5 g of powdered sample was each accurately weighed into 60 mL glass centrifuge tubes. Samples were extracted with organic solvent as follows: 2:1 (v/v) methanol/dichloromethane (3×), followed by 9:1 (v/v) dichloromethane/methanol (× 3). For each extraction, the tubes were sonicated for 10 min in an ultrasonic bath (room temperature). Extracts were separated from solid residues by centrifugation, and supernatants from each step were combined to give a total lipid extract (TLE). TLEs were concentrated to minimal volume under a gentle stream of high purity N_2_ gas. A portion each TLE was then subjected to acid methanolysis (0.5 N methanolic HCl, 60 °C–10 h), followed by silylation (99:1 N,O-Bis(trimethylsilyl)trifluoroacetamide /trimethylchlorosilane mixed with pyridine (1:1 v/v); 70 °C, 2 h). Aliquots of the derivatized samples were analyzed by gas chromatography/mass spectrometry (Agilent 5890 GC hyphenated to an Agilent 5975C Mass Selective Detector). The GC was equipped with a Gertsel programmable temperature vaporizer (70 °C ramped to 360 °C at a rate of 720 °C min^−1^) and a J&W 60 m capillary column (0.25 mm inner diameter, 250 µm film thickness). The GC temperature program was: 70 °C for 2 min, ramp at 10 °C min^−1^ to 130 °C, followed by a ramp to 300 °C at 4 °C min^−1^ and a final hold time of 20 min. The mass spectrometer was operated in electron impact ionization mode (70 eV), with a mass scan range from m/z 50 to 600. All solvents used were high-purity (OmniSolv) and all aqueous solutions were cleaned with dichloromethane prior to use, and procedural blanks were run to monitor background contamination.

## Supplementary Information

Supplementary Information.
