# Supplementary material for: Molecular preservation in mammoth bone and variation based on burial environment
Source: Sci Rep. 2021 Jan 29;11:2662. doi: 10.1038/s41598-021-81849-6 (PMC7846728; doi:10.1038/s41598-021-81849-6)
Supplement: Supplementary file 1 — Supplementary Information. [file 41598_2021_81849_MOESM1_ESM.docx]

Molecular preservation in mammoth bone and variation based on burial environment

Caitlin Colleary^1*^, Hector M. Lamadrid^2^, Shane S. O’Reilly^3^, Andrei Dolocan^4^, Sterling J. Nesbitt^5^

^1^ Department of Vertebrate Paleontology, Cleveland Museum of Natural History, Cleveland, OH 44106

^2^ Department of Geological Sciences, University of Missouri, Columbia, MO 65211

^3^ School of Earth Sciences, University College Dublin, Dublin 4, Ireland

^4^ Texas Materials Institute, University of Texas at Austin, Austin, TX 78712

^5^ Department of Geosciences, Virginia Tech, Blacksburg, VA 24061

*Corresponding author: ccolleary@cmnh.org

**Supplement**

**Table S1.** Amino acid fragments

| **Mass** | **Assignment** | **Loadings** (Figure 1B) | **Potential amino acids** | **Source** |
| --- | --- | --- | --- | --- |
| 30 | CH_4_N | 23 | G, A, S, P, V, T, L, E, M, F, R, Y | Orlando et al. 2013 |
| 44 | C_2_H_6_N | 34 | A, S, P, V, T, L, E, M, F, R, Y | Orlando et al. 2013 |
| 56 | C_3_H_6_N | 38 | A, S, P, V, T, L, E, M, F, R, Y | Orlando et al. 2013 |
| 59 | C_3_H_7_O | 18 | A, S, P, V, T, L, E, M, F, R, Y | Orlando et al. 2013 |
| 60 | C_2_H_6_NO | 28 | A, S, P, V, T, L, E, M, F, R, Y | Orlando et al. 2013 |
| 60 | C_3_H_8­_O | 28 | P, V, T, L, E, M, F, R, Y | Orlando et al. 2013 |
| 61 | C_2_H_5_S | 15 | M | Orlando et al. 2013 |
| 67 | C_4_H_5_N | 45 | P, V, T, L, E, M, F, R, Y | Assigned |
| 68 | C_4_H_6_N | 44 | P, V, T, L, E, M, F, R, Y | Orlando et al. 2013 |
| 69 | C_4_H_5_O | 47 | P, V, T, L, E, M, F, R, Y | Orlando et al. 2013 |
| 69 | C_2_H_3_N_3_ | 47 | R | Orlando et al. 2013 |
| 71 | C_3_H_3_O_2_ | 16 | A, S, P, V, T, L, E, M, F, R, Y | Orlando et al. 2013 |
| 72 | C_4_H_10_N | 42 | V, L, M, F, R, Y | Orlando et al. 2013 |
| 74 | C_3_H_8_NO | 17 | P, V, T, L, E, M, F, R, Y | Orlando et al. 2013 |
| 76 | C_5_H­_2_N | 48 | P, L, E, M, F, R, Y | Orlando et al. 2013 |
| 81 | C_4­_H_5_N_2_ | 40 | R | Orlando et al. 2013 |
| 81 | C_5_H_7_N | 40 | P, L, E, M, F, R, Y | Orlando et al. 2013 |
| 82 | C_4_H_6_N_2_ | 49 | R | Orlando et al. 2013 |
| 83 | C_5_H_9_N | 33 | P, V, L, E, M, F, R, Y | Orlando et al. 2013 |
| 85 | C_5_H_11_N | 46 | V, L, M, F, R, Y | Assigned |
| 87 | C_3_H_7_N­_2_O | 22 | R | Orlando et al. 2013 |
| 87 | C_3_H_9_N­_3_ | 22 | R | Orlando et al. 2013 |
| 88 | C_3_H_6_NO_2_ | 10 | A, S, P, V, T, L, E, M, F, R, Y | Orlando et al. 2013 |
| 91 | C_7_H_7_ | 26 | F, Y | Orlando et al. 2013 |
| 93 | C_3_H­_11_NO_2_ | 30 | V, L, M, F, R, Y | Assigned |
| 95 | C_5_H_5_NO | 39 | P, V, L, E, M, F, R, Y | Assigned |
| 96 | C_5_H_6_NO | 7 | P, V, L, E, M, F, R, Y | Assigned |
| 97 | C_4_H_3­_NO_2_ | 41 | P, V, T, L, E, M, F, R, Y | Assigned |
| 98 | C_4_H_4_NO_2_ | 31 | P, V, T, L, E, M, F, R, Y | Orlando et al. 2013 |
| 100 | C_4_H_10_N_3_ | 32 | R | Orlando et al. 2013 |
| 102 | C_4_H_8_NO­_2_ | 24 | P, V, T, L, E, M, F, R, Y | Orlando et al. 2013 |
| 103 | C_4_H_9_NO_2_ | 2 | P, V, T, L, E, M, F, R, Y | Assigned |
| 104 | C_4_H_10_NS | 21 | M | Orlando et al. 2013 |
| 107 | C_7_H_7_O | 37 | F, Y | Orlando et al. 2013 |
| 109 | C_7_H_11_N | 36 | F, Y | Assigned |
| 110 | C_5_H_8_N_3_ | 43 | R | Orlando et al. 2013 |
| 111 | C_5_H­_9_N_3_ | 29 | R | Assigned |
| 112 | C_5_H_10_N_3_ | 8 | R | Assigned |
| 113 | C_6_H_11_NO | 9 | L, F, R, Y | Assigned |
| 120 | C_8_H_10_N | 35 | F, Y | Orlando et al. 2013 |
| 122 | C_7_H_8_NO | 1 | F, Y | Assigned |
| 127 | C_5_H_11_N_4_ | 25 | R | Orlando et al. 2013 |
| 130 | C_9_H_8_N | 27 | F, Y | Orlando et al. 2013 |
| 131 | C_9_H_9_N | 19 | F, Y | Assigned |
| 138 | C_6_H_6_NO_2_ | 3 | L, F, R, Y | Assigned |
| 141 | C_6_H_9_N_2_O_2_ | 20 | R | Assigned |
| 152 | C_6_H_8_NO_2_ | 11 | L, F, R, Y | Assigned |
| 159 | C_10_H_11_N_2_ | 5 | N/A | Orlando et al. 2013 |
| 168 | C_8_H_10_NO­_3_ | 12 | Y | Assigned |
| 169 | C_8_H_11_NO_3_ | 13 | Y | Assigned |
| 171 | C_6_H_11_N_4_O_2_ | 6 | R | Assigned |
| 175 | C_9_H_5_NO_3_ | 4 | Y | Assigned |
| 177 | C_9_H_7_NO_3_ | 14 | Y | Assigned |

**Table S2.** Raman band assignments

| **Peak position range (cm-1)** | **Assignment** | **Literature References** |
| --- | --- | --- |
| 1620-1700 | Amide I | [1-3] |
| 1410-1480 | d(CH_2_), d(CN) | [1-3] |
| 1220-1300 | Amide III | [1-3] |
| 1070-1080 | Apatite carbonate substitution n_1_(CO_3_^2-^) | [1-6] |
| 1045-1050 | Apatite n_4_ (PO_4_^3-^) | [1, 5] |
| 1030-1045 | AGE/ALE | [2, 7] |
| 995-1005 | n Phe | [1, 2, 5] |
| 965-957 | Apatite n_1_ (PO_4_^3-^) | [1-6] |
| 900-920 | AGE/ALE, collagen | [2, 7] |
| 830-880 | AGE/ALE, collagen | [2, 7] |
| 800-820 | AGE/ALE | [2, 7] |
| 570-635 | Apatite n_4_ (PO_4_^3-^) | [1, 2, 5, 6] |
| 410-470 | Apatite n_2_ (PO_4_^3-^) | [1-6] |


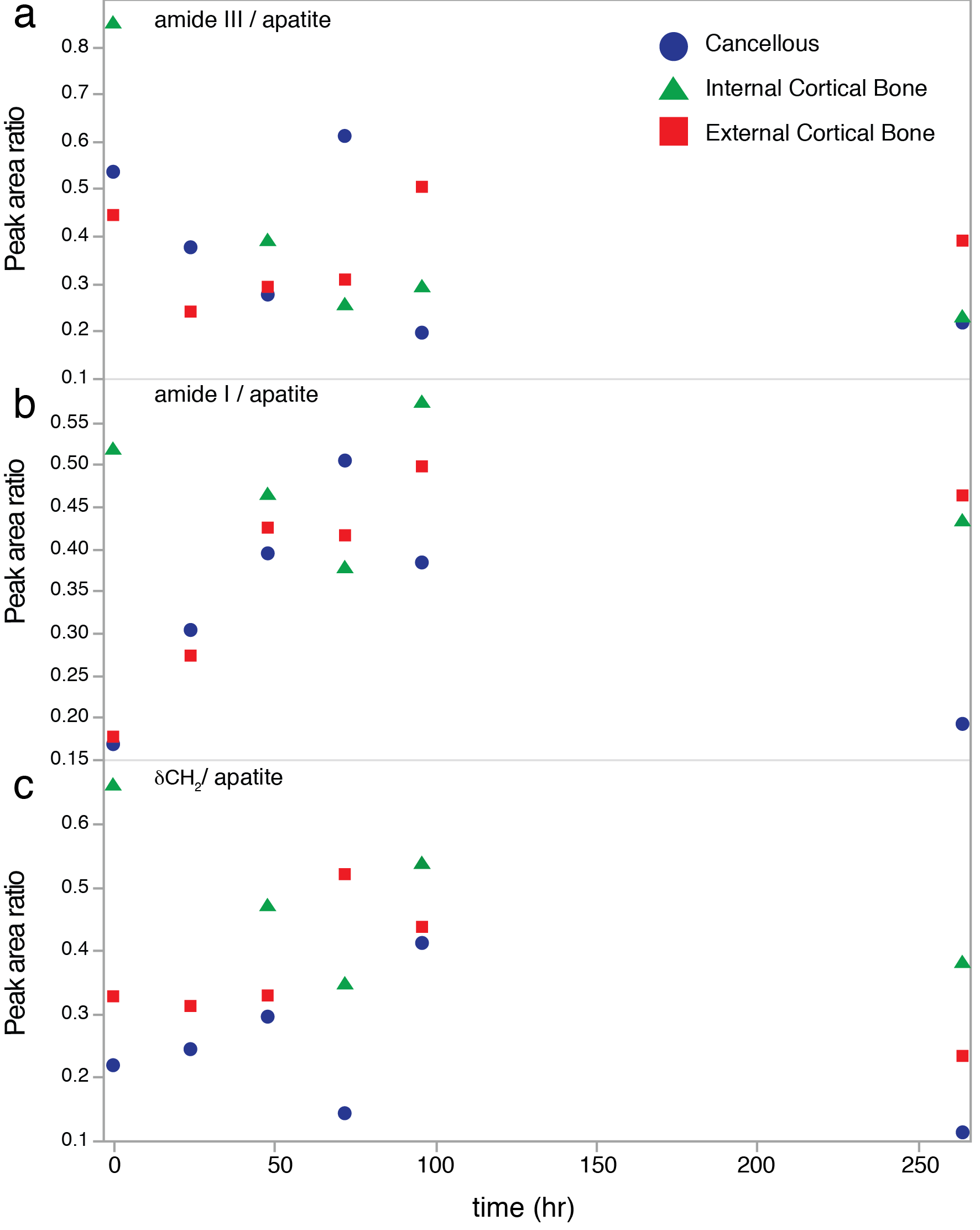


Figure S1. Spectral features of the amide bands from the maturation experiments. The ratios from the areas under the peaks of the amide bands and the apatite *v*1 peak indicate the degradation of the samples with time, following similar trends as those observed in the mammoth bones. We observed a systematic reduction of the ratios of the areas from three major peaks (amide III, amide II and C-C) and apatite peak. This ratio shows that progressively the organics diminish with time. The bone, however, does not degrade at the same rate. The three areas of interest, cancellous bone (blue), the internal part of the cortical bone (green) and the external part of the cortical bone (red), degrade at different rates controlled by the density of the bone from the less dense (cancellous) to the higher density of the bone in the external area of the cortical bone during the 11 days of the experiment.


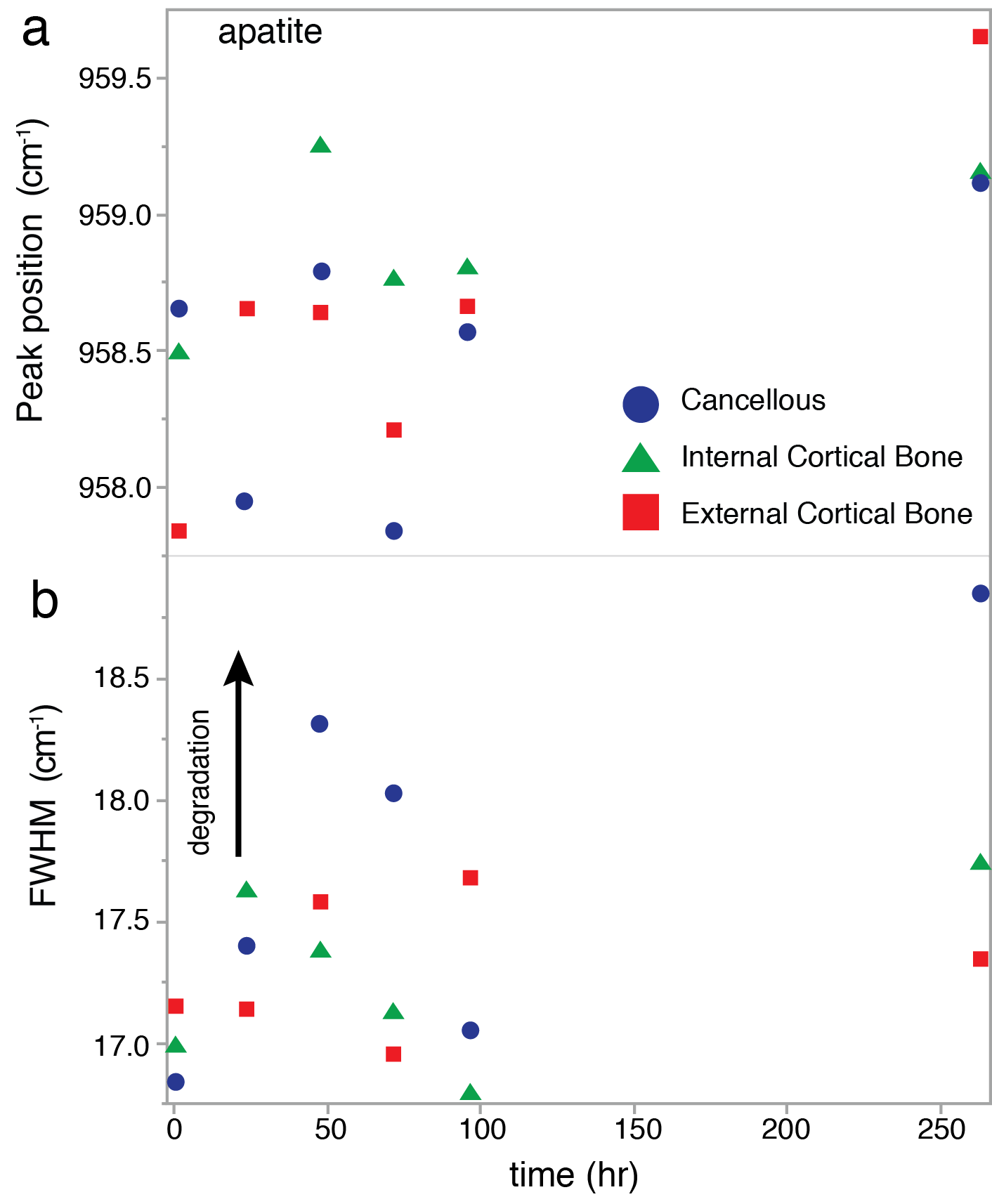


Figure S2. Raman spectral features of the apatite *v*1 band from three areas of interest (cancellous bone and the internal and external parts of the cortical bone from the modern elephant maturation experiments. The peak position and the width (full width at half maximum FWHM) from the three areas of interest show a systematic increase with time at 100 ºC. The increase in the FWHM is related to the degradation of the crystalline structure of bone.


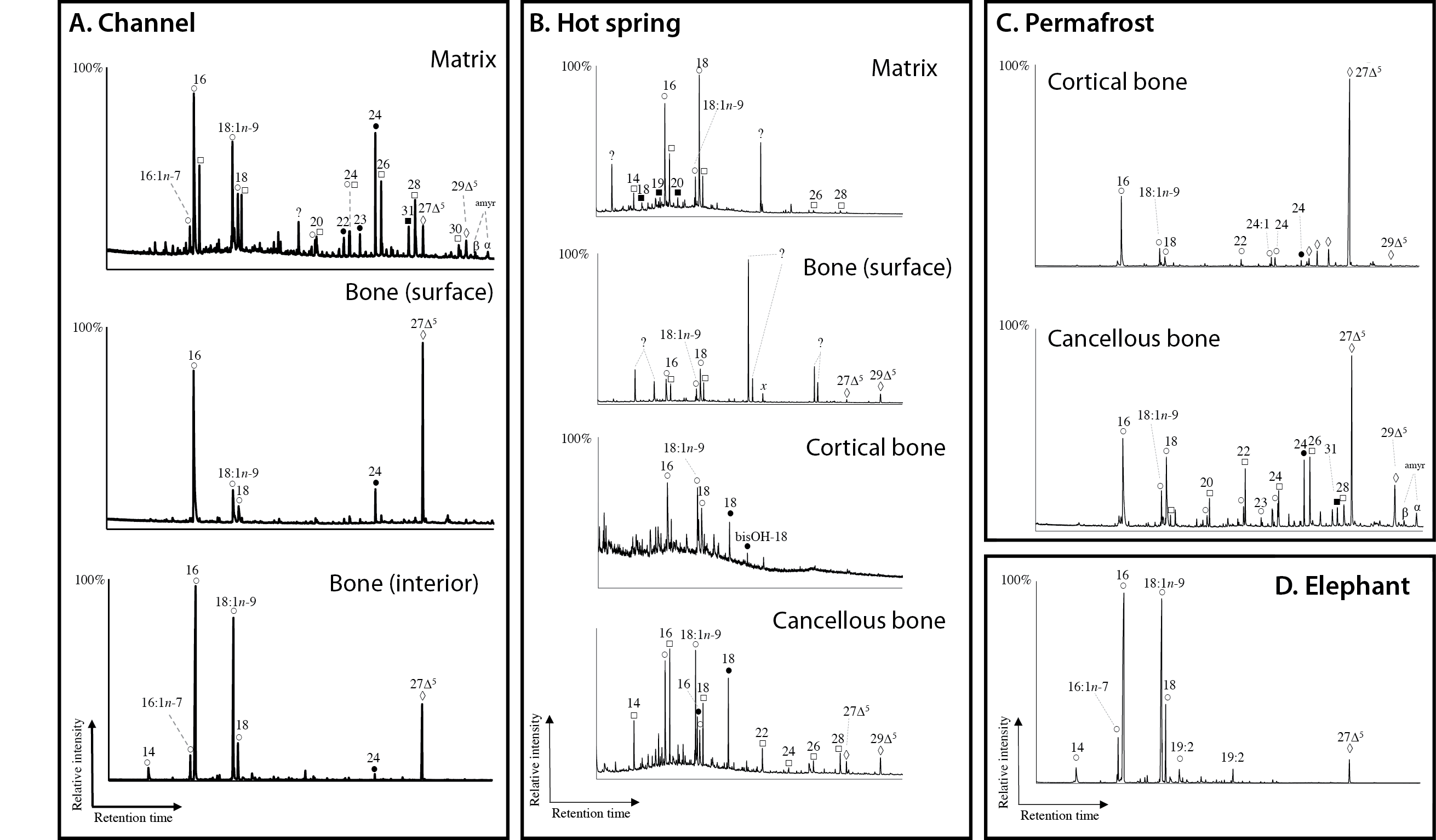


Figure S3.

Partial total ion chromatograms showing the detailed lipid distributions between different areas of the bone and matrix in A. channel deposit mammoth bone, B. hot spring mammoth bone, C. permafrost mammoth bone and D. extant elephant bone. ‘○’ are fatty acids, ‘●’ are 2-hydroxy fatty acids, ‘□’ are n-alkan-1- ols, ‘■’ are n-alkanes and ‘◊’ are sterols. ‘amyr’ corresponds to alpha and beta amyrin.

References

1. France, C.A., et al., *FT-Raman spectroscopy as a method for screening collagen diagenesis in bone.* Journal of archaeological Science, 2014. **42**: p. 346-355.

2. Brody, R.H., H.G. Edwards, and A.M. Pollard, *Chemometric methods applied to the differentiation of Fourier-transform Raman spectra of ivories.* Analytica Chimica Acta, 2001. **427**(2): p. 223-232.

3. Carden, A. and M.D. Morris, *Application of vibrational spectroscopy to the study of mineralized tissues.* Journal of biomedical optics, 2000. **5**(3): p. 259-269.

4. Wopenka, B. and J.D. Pasteris, *A mineralogical perspective on the apatite in bone.* Materials Science and Engineering: C, 2005. **25**(2): p. 131-143.

5. Awonusi, A., M.D. Morris, and M.M. Tecklenburg, *Carbonate assignment and calibration in the Raman spectrum of apatite.* Calcified tissue international, 2007. **81**(1): p. 46-52.

6. Antonakos, A., E. Liarokapis, and T. Leventouri, *Micro-Raman and FTIR studies of synthetic and natural apatites.* Biomaterials, 2007. **28**(19): p. 3043-3054.

7. Wiemann, J., et al., *Fossilization transforms vertebrate hard tissue proteins into N-heterocyclic polymers.* Nature Communications, 2018. **9**(1): p. 4741.
